# Unleashing the potential of digital pathology data by training computer-aided diagnosis models without human annotations

**DOI:** 10.1038/s41746-022-00635-4

**Published:** 2022-07-22

**Authors:** Niccolò Marini, Stefano Marchesin, Sebastian Otálora, Marek Wodzinski, Alessandro Caputo, Mart van Rijthoven, Witali Aswolinskiy, John-Melle Bokhorst, Damian Podareanu, Edyta Petters, Svetla Boytcheva, Genziana Buttafuoco, Simona Vatrano, Filippo Fraggetta, Jeroen van der Laak, Maristella Agosti, Francesco Ciompi, Gianmaria Silvello, Henning Muller, Manfredo Atzori

**Affiliations:** 1 Information Systems Institute, University of Applied Sciences Western Switzerland (HES-SO Valais), Sierre, Switzerland; 2 Centre Universitaire d’Informatique, University of Geneva, Geneva, Switzerland; 3 Department of Information Engineering, University of Padua, Padua, Italy; 4 Department of Measurement and Electronics, AGH University of Science and Technology, Krakow, Poland; 5 Department of Pathology, Ruggi University Hospital, Salerno, Italy; 6 Pathology Unit, Gravina Hospital Caltagirone ASP, Catania, Italy; 7 Department of Pathology, Radboud University Medical Center, Nijmegen, The Netherlands; 8 SURFsara, Amsterdam, The Netherlands; 9 MicroscopeIT, Wrocław, Poland; 10 Sirma AI, Sofia, Bulgaria; 11 Institute of Information and Communication Technologies, Bulgarian Academy of Sciences, Sofia, Bulgaria; 12 Pathology Unit, Cannizzaro Hospital, Catania, Italy; 13 Center for Medical Image Science and Visualization, Linkoping University, Linkoping, Sweden; 14 Medical Faculty, University of Geneva, Geneva, Switzerland; 15 Department of Neuroscience, University of Padua, Padua, Italy

**Keywords:** Pathology, Cancer imaging, Preventive medicine

## Abstract

The digitalization of clinical workflows and the increasing performance of deep learning algorithms are paving the way towards new methods for tackling cancer diagnosis. However, the availability of medical specialists to annotate digitized images and free-text diagnostic reports does not scale with the need for large datasets required to train robust computer-aided diagnosis methods that can target the high variability of clinical cases and data produced. This work proposes and evaluates an approach to eliminate the need for manual annotations to train computer-aided diagnosis tools in digital pathology. The approach includes two components, to automatically extract semantically meaningful concepts from diagnostic reports and use them as weak labels to train convolutional neural networks (CNNs) for histopathology diagnosis. The approach is trained (through 10-fold cross-validation) on 3’769 clinical images and reports, provided by two hospitals and tested on over 11’000 images from private and publicly available datasets. The CNN, trained with automatically generated labels, is compared with the same architecture trained with manual labels. Results show that combining text analysis and end-to-end deep neural networks allows building computer-aided diagnosis tools that reach solid performance (micro-accuracy = 0.908 at image-level) based only on existing clinical data without the need for manual annotations.

## Introduction

The digitalization of clinical histopathology workflows, along with the advancements of deep learning, is paving the way to Computer-Assisted Diagnostic (CAD) tools that can learn from clinical data without human intervention^1^, although several challenges remain.

Histopathology is the gold standard for cancer diagnostics^2^. It involves the examination of tissue sections to identify microscopic manifestations of diseases. Tissue samples are collected via biopsies or surgical resections and then prepared to undergo microscopic examination by a pathologist. The manual analysis is a time-consuming task lasting up to one hour per image^3^. However, heterogeneous tissue morphologies, arbitrary selection of the tissue regions to analyze in detail and subjective evaluation of findings^4^ generally lead to a low inter-pathologist agreement on the diagnosis^5–7^.

The processing and analysis are usually performed with limited digital assistance in clinical practice, even though digital pathology is becoming increasingly common^8^. Digital pathology involves acquiring and managing digitized tissue specimens, called whole slide images (WSI). Whole slide scanners usually acquire images with a high optical magnification of x20–40, resulting in a spatial high-resolution of 0.25–0.5 μm per pixel. WSIs are generally stored in a multi-scale format, allowing pathologists to visualize different details of the images during the analysis, from the lowest to the highest magnification levels. Pathological findings, including observations from WSI analysis, are usually described in a pathology case report. Even though synoptic reports (including specific data about the patient in a structured format) are expected to become increasingly common^9^, semi-structured free-text reports are still the standard in clinical settings^10^. Semi-structured reports include several fields, such as the type of tissue specimen, the findings identified during the analysis, an early diagnosis and the patient’s anamnesis. The number of hospitals digitizing WSIs is increasing^11–13^, allowing the collection of thousands of images and diagnoses.

Computational pathology is a recent domain centered on computer-assisted diagnosis tools to analyze digital pathology images automatically. Convolutional neural networks (CNNs) have emerged as the state-of-the-art method to solve several computational pathology tasks, reaching high performance. However, despite an increasing number of methods, applications, and scientific findings, the full potential of digital clinical pathology data is still not reached and several challenges are still open. First, CNNs usually need large datasets for training models that can deal with the high data variability of clinical practice^14^. Second, fully supervised approaches, that provide the highest performance in computational pathology, require pixel-wise annotations^15^ that are challenging to obtain in medical contexts as they are resource- and time-consuming^16,17^. Third, WSIs are challenging to manage and fit into memory, even with modern hardware^18,19^, since they are usually very large. Thus, splitting the WSIs into patches is a common and required practice, sometimes leading to biases due to the loss of spatial relationships between the patches. Finally, WSIs can be highly heterogeneous in stain variations due to the lack of standardization in tissue preparation and acquisition across images and centers^20,21^. Stain heterogeneity leads to low model generalization on data acquired from heterogeneous medical contexts that may include different stain variations than those included in the data used to train the models.

In recent years, weakly supervised learning approaches have emerged to target some of these challenges^16,22–24^. Weakly supervised learning approaches use global (weak or image-level) annotations instead of local (pixel-wise) annotations. Global annotations usually refer to the whole image, even though they are usually derived from a specific and small sub-region of the image. For instance, a WSI would likely be labeled as containing “cancer”, even if the cancerous tissue is present only in 1–2% of the entire image. Therefore, weakly supervised CNNs require training datasets bigger than fully supervised approaches to reach comparable performance.

On the other hand, global annotations present the potentially groundbreaking advantage that they can be inferred from reports, often provided together with WSIs. Nevertheless, up to now, medical experts were needed to extract weak labels from the report in most cases.

Campanella et al.^16^ achieved excellent cancer classification performance (AUC = 0.986), relying on weak annotations and weakly supervised methods. They trained a CNN with a Multiple Instance Learning (MIL) framework to classify WSIs into two classes (cancer vs. non-cancer) with a dataset including over 30,000 WSIs of prostate, breast, and skin tissue slides. Despite the high performance, this work only partially highlights the potential of digital pathology data due to two main reasons. First, the weak labels were manually provided by pathologists, after a time-consuming WSI analysis, or automatically retrieved, thanks to the structured nature of the Laboratory Information System (LIS), where the reports are stored with predefined and structured fields that allow to retrieve the concepts in the diagnosis easily. Unfortunately, most LISs do not have a structured nature and deal with noisy and heterogeneous free-text reports. Therefore, the global diagnosis for the images can, in most cases, only be inferred from the pathology reports with the intervention of medical experts. The manual annotation of reports is faster than pixel-wise annotation of images but it is still time-consuming procedure, thus limiting the usability of clinical workflow data to train models at a very large scale. Second, Campanella et al. considered only two classes. The binary setup might be due to the study’s novelty or the methodology used to perform the annotations. However, it still does not correspond well to the potential of clinical digital pathology workflows, where several classes and diagnostic perspectives are presented in the report paired to a tissue slide.

This paper proposes and evaluates an approach to alleviate the limitations preventing fully exploiting digital clinical pathology for training-assisted diagnosis tools. The proposed approach includes a Natural Language Processing (NLP) pipeline to automatically analyze free-text reports and a computer vision algorithm trained with weak annotations to classify images. The NLP pipeline automatically extracts semantically meaningful concepts from free-text diagnosis reports to be used as weak labels for training an image classifier. The implementation of the approach can be changed and modified, allowing to adopt different techniques that vary depending on the characteristics of the problem to solve and on the state-of-the-art algorithm advancement. The approach is tested on digital pathology colon data, completely bypassing the need for human and unleashing the potential of data acquired in clinical workflows. To demonstrate the reliability of automatically generated weak labels for training, the image classifier is compared with the same image classifier architecture, trained using manual weak labels.

**Fig. 1:**
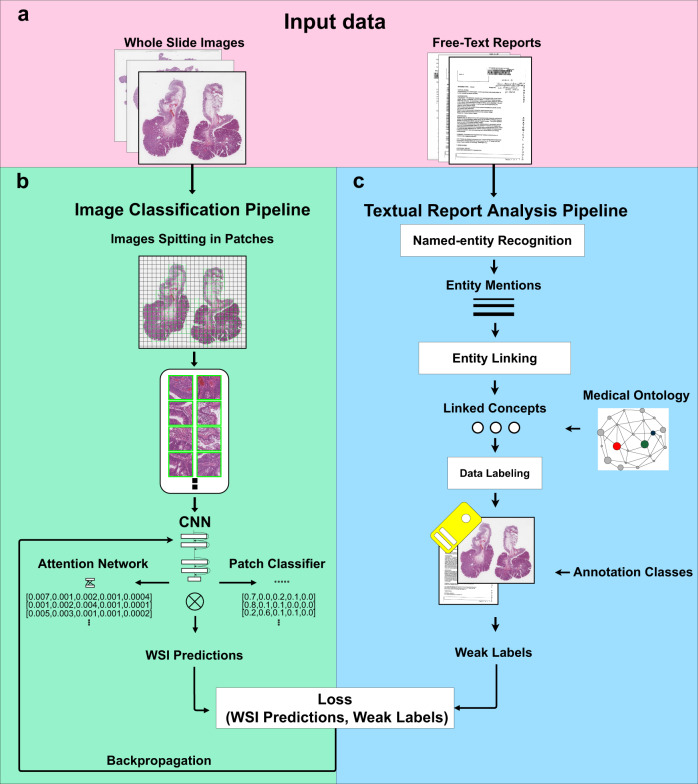
Overview of the analysis pipeline. **a** Input data from the clinical workflow (pink background) including WSIs (left) and the corresponding free-text pathology reports (right). **b** Image Classification pipeline (green background) includes WSI pre-processing and image classification. The WSI pre-processing involves the image splitting into patches from magnification x10, with a size of 224×224 pixels to fit the pre-trained ResNet34 architecture. The image classification involves a CNN, trained using a Multiple Instance Learning algorithm. The CNN includes a frozen ResNet34 backbone (convolutional layers with ImageNet weights) that produces feature vectors with 512 elements per patch; an embedding layer to reduce the feature vector to 128 elements; a classifier that produces predictions at patch-level; an attention network, that learns to identify relevant patches and aggregates the patch-level predictions to have a global WSI-prediction. **c** The textual report pipeline automatically analyzes pathologist reports, to identify meaningful concepts to be used as weak labels for the CNN.

Figure 1 describes the two components of the pipeline.

The extraction of meaningful concepts from pathology reports relies on the Semantic Knowledge Extractor Tool (SKET). SKET is an *unsupervised* hybrid knowledge extraction system that combines a rule-based expert system with pre-trained machine learning models to extract labels from free-text reports.

Image classification relies on a Multiple Instance Learning (weakly supervised framework) CNN. The CNN is trained with the weak labels provided by SKET. The proposed CNN makes predictions at patch-level (multiclass) and aggregates them using an attention pooling layer^22,23^, to have WSI-level predictions (multilabel). The CNN produces multilabel predictions reflecting the pathology report nature: the analysis of the images may highlight several findings in the same sample. Usually, in scientific literature, the analysis of WSIs with multiple instance learning involves binary^16,24,25^ or multiclass^22,23,26^ classification, often with the most dangerous findings (e.g. cancer) identified as weak labels. Adopting a network that makes multilabel predictions allows to better approximate the nature of tissue samples.

The proposed approach is trained using colon WSIs and reports provided by the Catania cohort (Azienda Ospedaliera Cannizaro and Gravina Hospital Caltagirone ASP, Catania, Italy) and the Radboud Medical University Center (Radboudumc, Nijmegen, The Netherlands) and tested on private and publicly available datasets using five classification classes. The hospitals provided the reports and the WSIs without any manual data curation or expert supervision, thus representing an ideal scenario for testing the proposed approach.

Colon is chosen as a use case due to its high impact on society and the difficulty of diagnosing it. Colon is the fourth most commonly diagnosed cancer in the world^27^ with a 75% increase predicted by 2040 for both genders and a broad range of ages^28^. The diagnosis of colon cancer is problematic because it requires the identification of malignant polyps^27^ (agglomerations of cells protruding from the colon surface) and can include several classes, namely adenocarcinoma, high-grade dysplasia (HGD), low-grade dysplasia (LGD), hyperplastic polyp and normal.

## Results

### Data

A total of 15,601 colon histopathology images (4419 paired with the corresponding report from clinical workflows and 11,888 from publicly available datasets) were used in this work, with focus on five classes. A detailed description of data is provided in Table 1, while the Method section provides further details on data characteristics.

**Table 1:** Overview of the dataset composition.

	Class					
Source	Cancer	High-grade dysplasia	Low-grade dysplasia	Hyperplastic polyp	Normal	Total images
Training dataset: automatic weak labels (SKET)						
Catania	422	464	630	251	462	1704
Radboudumc	189	119	434	493	1000	2065
Total	611	583	1064	744	1462	3769
Training dataset: manual weak labels (ground truth)						
Catania	379	454	529	181	438	1704
Radboudumc	188	94	453	428	1048	2065
Total	567	548	982	609	1486	3769
Private testing datasets						
Catania	52	44	54	23	79	227
Radboudumc	50	23	92	62	219	423
Total	102	67	146	85	298	650
Public testing datasets						
GlaS^36^	91	0	0	42		133
CRC^37^	69	0	0	71		140
UNITOPATHO^31,32^ (sections)	0	1370	5804	545	950	8669
UNITOPATHO^31,32^ (WSI)	0	46	184	41	21	292
TCGA-COAD^33^	50	0	0	0	0	50
Xu^38^	355	0	0	0	362	717
AIDA^34^	31	4		1	65	101
IMP-CRC^35^	268		547	271		1086
**Total**						11888

The dataset includes colon images and reports from digital pathology workflows (Catania and Radboudumc) and publicly available datasets. The dataset is split into training (upper part) and testing (lower part). The training dataset is labeled using automatically extracted weak labels provided by SKET (upper part) and the ground truth of manually annotated weak labels (central part). The training partition includes data from Catania and Radboudumc, used to train the CNN with a 10-fold cross-validation approach and evaluate the approach comparing its performance after training with automatically extracted labels and manually-created labels. The test partition (lower part) includes data from Catania and Radboudumc and data from public datasets. Public datasets are in some cases labeled with different classes than those employed in this work. In such cases, classes are mapped to the five considered ones via aggregation. The task proposed in the paper is a multilabel classification problem, therefore the sum of the rows can differ from the total number of images. Furthermore, SKET weak labels can include mislabeled samples, therefore the sum of the rows can differ between the automatic and the manually-created weak labels, whereas, the total number of images is the same.

### Weak labels for images can be extracted from free-text diagnostic reports

High quality semantically meaningful concepts (usable as labels for whole slide images) can be extracted from diagnostic reports without human interaction, allowing to replace manual annotations created by experts on large scale datasets and to drastically reduce time and effort required for data annotation.

The performance of SKET (the tool targeting label extraction) is evaluated on 3769 diagnostic reports corresponding to the data used to train and validate the CNN (1704 from Catania, 2065 from Radboudumc).

Experts manually labeled the reports for ground truth creation purposes, according to the five classes described above. The task is a multilabel classification problem, because each report can be annotated with one or more classes.

Table 1 reports the class distribution of the reports (the upper part includes the weak labels provided by SKET, the central part includes the manually annotated weak labels) for both hospitals. Dataset class imbalance reflects a realistic scenario, where certain conditions (e.g., normal samples) occur more often than others in clinical routine. Free-text reports are not curated before the execution of SKET. In order to deal with multilingualism, reports in Italian (Catania) and Dutch (Radboudumc) are translated to English using the pre-trained MarianNTN^29^ neural machine translation models, a Transformer-based^30^ encoder-decoder architecture with six layers in each component. SKET is evaluated using micro-accuracy and weighted macro F1-score. On the Catania data, SKET achieves a 0.933 micro-accuracy and a 0.867 weighted macro F1-score; on the Radboudumc data, SKET achieves a 0.950 micro-accuracy and a 0.883 weighted macro F1-score. The results — further described in the “SKET limitations” paragraph, Methods section — show the effectiveness of SKET on both datasets.

By automatically analyzing pathology reports to extract weak annotations, SKET saves an important amount of time in annotation effort. An expert requires 30 seconds on average to annotate a diagnostic report (as the average time evaluated during the report annotation process), whereas SKET annotates more than three reports (3.19) per second. Therefore, SKET saves 95.7% of the time required for a pathologist to annotate a report. Projecting the time to annotate data on a number of reports comparable to Campanella et al. (over 30'000 WSIs, the largest WSI dataset ever used) and assuming that the human expert never stops, the time needed would be to over 250 h of human work (without breaks), while the NLP pipeline needs about 2.5 h.

The weak labels automatically extracted by SKET from the diagnostic reports of Catania and Radboudumc hospitals match the manual ground truth labels with high accuracy. Besides, SKET drastically reduces the time required to perform report annotations.

### The CNN trained with automatically generated labels obtains high performance on private data WSI-level classification

The CNN trained with weak labels automatically generated from reports is highly effective for multilabel WSIs classification. The CNN is evaluated at WSI-level using an internal test partition, including WSIs from Catania and Radboudumc with human-created report annotations.

The CNN is trained with a MIL framework, based on multiclass patch-level predictions and an attention network to aggregate the multilabel predictions at the WSI-level. It classifies five classes (cancer, high-grade dysplasia, low-grade dysplasia, hyperplastic polyp and normal). The CNN is trained using concepts extracted from diagnostic reports by SKET as weak labels, so without any human pixel-wise annotation.

The CNN, trained with the automatically extracted weak labels, is compared with a CNN including the same architecture but trained using manually created weak labels on the same images. The Wilcoxon Rank-Sum test (*p* value < 0.05) is adopted to verify if the performance difference is statistically significant.

The CNN reaches micro-accuracy = 0.908 ± 0.005 (respectively 0.911 ± 0.004 on Catania and 0.906 ± 0.005 on Radboudumc), macro weighted F1-score = 0.769 ± 0.018 (respectively 0.797 ± 0.011 on Catania and 0.744 ± 0.020 on Radboudumc) and there is no statistically significant difference between using automatic and manual (ground truth) weak labels for training.

**Fig. 2:**
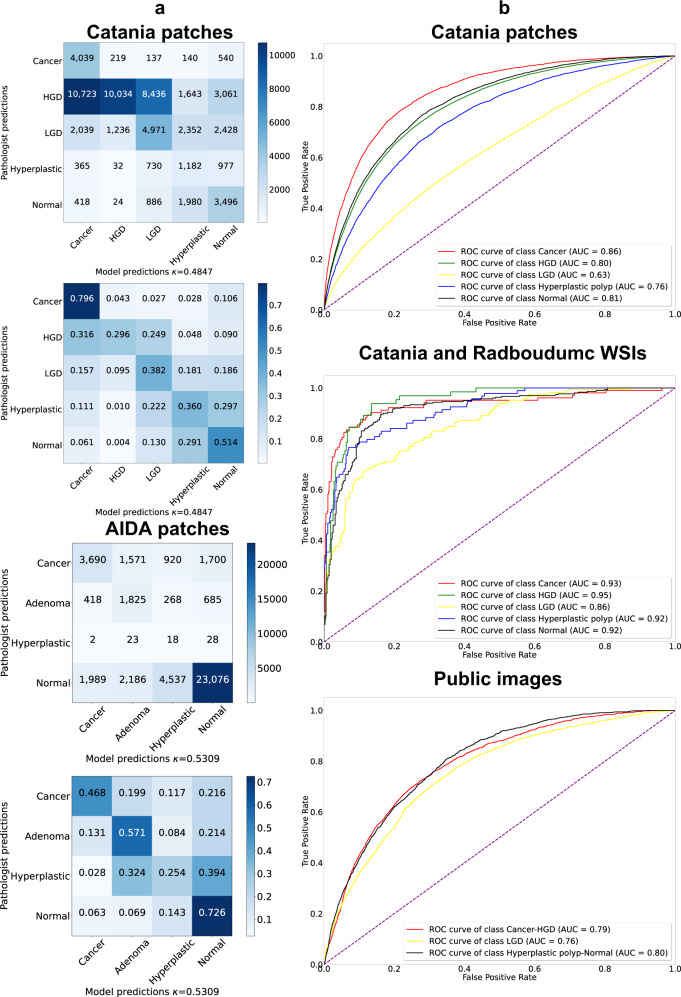
Quantitative evaluation of the classification models at patch- and WSI-level. **a** Confusion matrices of the CNN models that reach the highest performance in the patch-level classification. The matrices include the raw and the normalized values. The matrices are reported for Catania (upper part) and AIDA (lower part). The AIDA dataset includes a class called dysplasia, instead of high-grade and low-grade dysplasia. The ground truth and the predictions are mapped into the dysplasia class. **b** ROC curves of the CNN models for the patch-level classification (Catania), the WSI-level classification (Catania) and the image-level classification (publicly available data). In the latter sub-Figure, the predictions are aggregated to match the different annotations across publicly available datasets.

The relevance of the result is related to the multilabel nature of the WSI classification problem and to the absence of human involvement into the training process. Figure 2b shows the ROC curve for WSI-level classification on private data.

Single class classification performance shows results for all the classes, with AUC over 0.92 for each class, except low-grade dysplasia, 0.85. The performance obtained on data from Catania is slightly higher compared with the one obtained in Radboudumc data.

### The CNN trained with automatically generated labels generalizes well on publicly available datasets

The CNN trained with weak labels automatically generated from reports demonstrates the capability to generalize well on heterogeneous images, from various medical centers.

The publicly available test partition includes 11,888 images collected from seven publicly available datasets. The test partition includes WSIs (UNITOPATHO^31,32^, TCGA-COAD^33^, AIDA^34^, IMP-CRC^35^) and cropped sections of WSIs (GlaS^36^, CRC^37^, UNITOPATHO^31,32^, Xu^38^). Sections of WSIs are treated as WSIs, since they are provided with labels referring to the whole image. The images collected from publicly available sources may be annotated with slightly different labels; therefore the predictions made by the model are aggregated to match the original labels (as shown in Table 1).

The CNN reaches good performance on publicly available datasets (F1-score over 0.72 for each the binary problems and over 0.58 for each of the multiclass problems, Table 2). The performance obtained in some publicly available datasets is comparable to the results on the private data test set.

**Table 2:** CNN performance overview.

Performance at WSI-level (private data)				
Dataset	Micro-accuracy (SKET labels)	Micro-accuracy (GT labels)	Weighted F1-score (SKET labels)	Weighted F1-score (GT labels)
Catania	0.911 ± 0.004	0.918 ± 0.006	0.797 ± 0.011	0.807 ± 0.020
Radboudumc	0.906 ± 0.005	0.909 ± 0.008	0.744 ± 0.020	0.758 ± 0.025
Private data	0.908 ± 0.005	0.912 ± 0.006	0.769 ± 0.015	0.779 ± 0.019
Performance on publicly available images				
Dataset	Accuracy (SKET labels)	Accuracy (GT labels)	Weighted F1-score (SKET labels)	Weighted F1-score (GT labels)
GlaS^36^	0.745 ± 0.059	0.745 ± 0.065	0.717 ± 0.050	0.750 ± 0.066
CRC^37^	0.876 ± 0.014	0.856 ± 0.024	0.878 ± 0.019	0.855 ± 0.024
UNITOPATHO^31,32^ (single sections)	0.549 ± 0.025	0.543 ± 0.026	0.590 ± 0.015	0.591 ± 0.020
UNITOPATHO^31,32^ (WSIs)	0.750 ± 0.022	0.770 ± 0.025	0.723 ± 0.024	0.764 ± 0.023
TCGA-COAD^33^	0.862 ± 0.051	0.868 ± 0.093	0.925 ± 0.029	0.927 ± 0.056
Xu^38^	0.717 ± 0.053	0.728 ± 0.038	0.677 ± 0.084	0.725 ± 0.041
AIDA^34^	0.743 ± 0.046	0.760 ± 0.030	0.744 ± 0.047	0.752 ± 0.026
IMP-CRC^35^	0.706 ± 0.035	0.678 ± 0.048	0.707 ± 0.033	0.682 ± 0.048

Results for the performance of the CNN on WSI-level classification task for the Catania and Radboudumc datasets (upper part) and for the classification of images from publicly available datasets (lower part). The performance at WSI-level is evaluated with micro-accuracy and weighted F1-score. For each classification type, the average and the standard deviation (of the models involved in the k-fold cross-validation) are reported for each metric, including cumulative results for each dataset. The performance is reported for the CNNs trained using the automatically generated weak labels (SKET labels) and the manually created ground truth weak labels (GT labels).

The obtained results are encouraging, as they show that the CNN can generalize – albeit with slightly lower performance than for private data – to external heterogeneous datasets, guaranteeing competitive performance on external datasets. More details on the data and on the class matching performance are provided in the Method, ‘Publicly available datasets class matching’ section.

### The CNN trained with automatically generated labels is robust to automated report labeling errors

Despite some limited performance difference, the CNN trained with weak labels automatically generated from reports shows robustness to errors introduced by such an automatic extraction process.

To validate this outcome, the CNN predictions of the models — trained with automatic and manual weak labels, respectively — are evaluated on those WSIs used to train and validate the CNN that are mislabeled by SKET. A mislabeled sample includes one or more classes generated by SKET that do not correspond to the multilabel ground truth. SKET mislabeled 25% of the WSIs (421 of 1704) from Catania and 15% of the WSIs (306 of 2065) from Radboudumc (i.e. a mislabeled sample means that at least one label related to a sample is not well predicted). The results are summarized in Table 3. The results show a limited difference in the average values of the CNNs trained with automatically and manually generated weak labels, for both micro-accuracy and weighted F1-score.

**Table 3:** Results of the CNN on the five classes WSI classification task on the SKET mislabeled samples, considering both the models trained with automatically and manually labeled WSIs.

Dataset	Micro-accuracy (SKET labels)	Micro-accuracy (GT labels)	Weighted F1-score (SKET labels)	Weighted F1-score (GT labels)
Catania	0.817 ± 0.037	0.831 ± 0.022	0.579 ± 0.032	0.599 ± 0.039
Radboudumc	0.835 ± 0.008	0.851 ± 0.014	0.571 ± 0.027	0.627 ± 0.036

The difference is not statistically significant on the Catania data, while it is for Radboudumc with *p* value = 0.019 for micro-accuracy and *p* value = 0.019 for weighted F1-score, respectively.

Thus, the noise introduced by SKET limitedly affect the training process of the CNN on Radboudumc data while the performance obtained by training the CNN with automatic labels is as effective as the one obtained with manual labels on the Catania data, demonstrating robustness of the CNN-based approach to mislabeled WSIs.

### The CNN trained with automatically generated labels leads to moderate patch-level classification

The CNN trained with weak labels automatically generated from reports reaches moderate performance on the patch-level classification.

Patch-level classification is a challenging task, considering that the model was trained without any pixel-wise annotation, optimizing image-level predictions via Multiple Instance Learning instance-based framework.

Table 4 and Fig. 2 summarize the results. Table 4 includes the performance obtained at patch-level, using pixel-wise annotated patches from the test partition of the Catania dataset and the AIDA^34^ datasets.

**Table 4:** Results for the performance of the CNN on the five classes patch-level classification task. The performance is evaluated with Cohen’s κ-score, reporting the average and the standard deviation of the models involved in the k-fold cross-validation. The performance is reported for the CNNs trained using the automatically generated weak labels (SKET labels) and the manually created weak labels (GT labels).

Performance at patch-level		
Dataset	κ-score (SKET labels)	κ-score (GT labels)
Catania	0.432 ± 0.027	0.413 ± 0.029
AIDA	0.482 ± 0.018	0.475 ± 0.008

The model obtains moderate performance (i.e. by definition κ-score^39^ between 0.40 and 0.60) without any information about the single patches used during the training: Cohen κ-score = 0.432 ± 0.027 on the Catania test partition (58,286 patches) and Cohen κ-score = 0.482 ± 0.018 on the AIDA publicly available images (43,036 patches). There is no significant difference between the results obtained using manual annotations and automatically generated annotations for training the CNN.

Figure 2a shows the confusion matrices of the CNN in patch-level classification and the ROC curve for all the presented tasks. The reported confusion matrices (raw and normalized values) refer to the model with the highest performance in patch-level classification. On the Catania data, the model classifies cancer and normal very effectively (over half of the samples are well classified), whereas it shows lower performance for the other classes, especially for the high-grade dysplasia class. Noteworthy, the misclassification mostly involves similar classes. For example, several high-grade dysplasia patches are misclassified with cancer and low-grade dysplasia, two classes including deformed glands, a tissue morphology present also in high-grade dysplasia patches; hyperplastic polyp patches are misclassified with low-grade dysplasia or normal, two classes including well-shaped glands, a tissue morphology present also in hyperplastic polyp patches. In particular, since a hyperplastic polyp is considered a not dangerous abnormality in the short-term^40^, the two classes are aggregated into one in some works^41^. On the AIDA data, the model classifies most of the classes with good performance. The results are relevant because they show that the model reaches high performance in normal patches classification, which represents the most represented condition in digital pathology workflows, especially in screening analysis. The ROC curve for patch-level classification of Catania data (first sub-Fig. 2b), shows the good performance reached in cancer and normal tissue classification.

### Correspondence of the CNN latent space to meaningful classes

The latent space of the CNN trained with weak labels automatically generated from reports shows a good separation between normal tissue and cancer morphologies

**Fig. 3:**
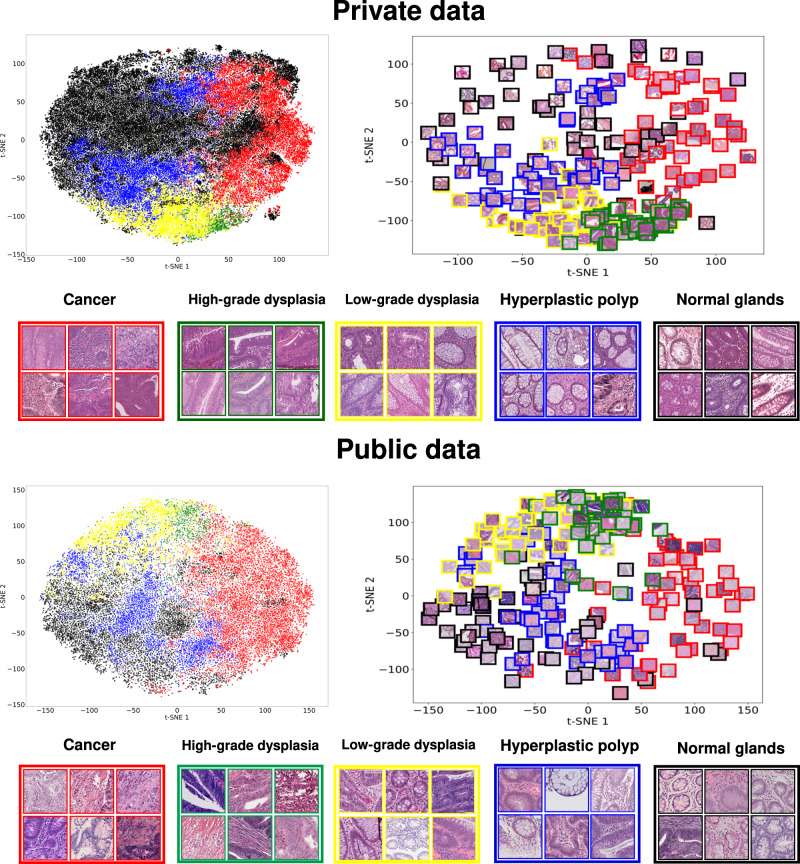
Qualitative model evaluation. The graphs include the feature embedding produced by the CNN, projected in two dimensions with t-SNE, for data coming from the private test partition (Private data, above) and the publicly available datasets (Public Data). The embeddings are represented as dots on the left side of the Figure, to show the class distribution, and as randomly selected patches on the right side of the Figure. The classes are: cancer (red), high-grade dysplasia (green), low-grade dysplasia (yellow), hyperplastic polyp (blue) and normal (black).

The CNN learns a feature representation of the data that allows to separate regions including tissue morphologies, which are linked to different classes. Figure 3 shows the patches in the latent space of the Catania and Radboudumc test partition (upper part) and of the publicly available datasets (lower part).

The latent space includes a two-dimensional representation of the samples (the output of the CNN embedding layer, 128 elements per patch) and is created with t-distributed stochastic embedding (t-SNE)^42^. For both the data sources, the left part of Fig. 3 includes dots representing the patches, while the right part shows some randomly selected patches corresponding to the dots. Each dot is colored with the predicted class: red (cancer), green (high-grade dysplasia), yellow (low-grade dysplasia), blue (hyperplastic polyp) and black (normal).

In both datasets, the patches predicted with the same class are projected in the same specific region of the space, even though overlapping exists in the border. The fact that the classes involved in this paper show similar morphologies may explain regions overlapping. For example, classifying the hyperplastic polyps and normal involves identifying well-shaped glands, while dysplasia is characterized by the deformation of glands and the neighboring stroma.

On private data latent space, it is possible to identify a region, on the right, including poorly defined glands and infiltrated stroma, linked to cancer patches (red); while on the left, it is possible to identify a region including healthy tissue, such as well-defined glands. A large variety of glands morphologies is placed between these two macro-regions, from poor definition (the green region, including high-grade dysplasia patches) to well-shaped glands (the blue region, including hyperplastic polyp patches). Remarkably, the same structure can be identified in the latent space of the public data, despite the heterogeneity: two macro-regions with cancer and normal patches and smaller regions with patches including different gland morphologies, linked to dysplasias or hyperplastic polyps.

Furthermore, another point to stress involves the fact that the regions are stain invariant, since it is possible to identify heterogeneous stains in the same region. This characteristic may be explained considering the CNN pre-training (presented in Method) includes a H&E-invariant CNN training^43^.

### CNN attention model identifies relevant tissue regions

The highest attention values of the CNN trained with labels automatically extracted from reports are in regions which are relevant to the predicted classes.

**Fig. 4:**
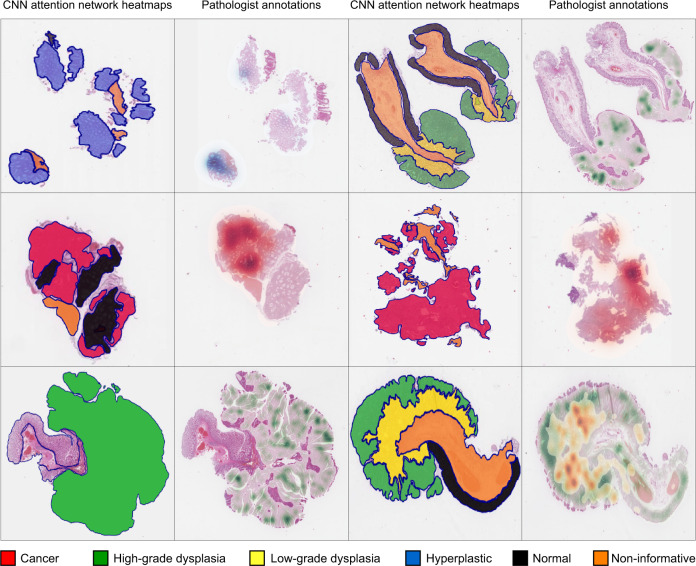
Heatmaps. A few examples of heatmaps, generated with the attention network’s weights, compared with manual pixel-wise annotations made by pathologist. Each couple of examples includes a heatmap (on the left) and the corresponding pathologist pixel-wise annotations (on the right). The highlighted regions within the heatmaps represent the ones where model assigned the highest importance for the global diagnosis. The comparison of the heatmaps and the annotations shows that the attention network gives greater importance to regions including relevant patches.

Currently, the attention network represents the state-of-the-art pooling layer used to aggregate the predictions at the patch-level to have predictions at WSI-level^22^. The network weighs the patches for each class so that the ones with the highest values of attention contribute more to global predictions. In Fig. 4, the weights assigned by the network to the patches of the internal test partition are visualized as heatmaps. The heatmap analysis shows that the regions where the attention model focuses most for each class include patches annotated with the corresponding class by pathologists in pixel-wise annotations. Therefore, the attention network gives greater importance to regions including relevant patches, leading the CNN to predict the correct global diagnosis.

## Discussion

This paper presents an approach to limit the need for human-made annotations to train computer-assisted diagnostic tools in digital pathology. The approach includes two components, represented by SKET and a CNN, allowing to automatically extract meaningful semantic concepts from pathologist reports and to use them as weak labels for high-resolution clinical pathology images, without any human supervision.

The approach is evaluated by training on private data (colon reports and WSIs provided by hospitals) and testing on an unseen subset of the private data and on external publicly available data. Private and publicly available data are highly heterogeneous, collected from nine different sources. Private data include over 3,700 WSIs with the corresponding reports in two languages (Italian and Dutch), while publicly available data include 11,888 images. The results show that it is possible to use clinical free-text reports and images to train computer-assisted diagnostic tools without any supervision, in the context of digital pathology. By applying well-established and reproducible methods, the proposed approach provides a solid baseline at WSI-level in highly heterogeneous private and publicly available datasets. This study has a remarkable implication: no human intervention is needed to annotate free-text clinical pathology data to train computer-aided diagnosis systems. This result has three main consequences.

The first consequence is a potential breakthrough in the digital pathology domain. Since it is possible to overcome the need for human intervention to annotate images and reports, it is also possible to exploit exascale datasets coming from heterogeneous pathology workflows, unleashing the full potential of digital pathology. The fact that the presented approach does not need any human annotation removes all the constraints related to data annotation when free-text diagnostic reports are available, allowing the collection of massive clinical datasets (including hundreds of thousand WSIs) for training computer-aided diagnosis systems on a variety of concepts presented in routine reports. Data processing without curation shows that it is possible to exploit data from several centers, overcoming the limitations of standardization in image format, text report format, and image processing systems. Increasing the number of centers can improve the performance of the algorithms in terms of capability to deal with image heterogeneity, allowing researchers to collect big datasets to train robust tools with limited effort and triggering a virtuous circle in the computational pathology domain.

The second consequence is related to the developed computer-assisted diagnosis models that can (after further improvement of the performance) reduce the time needed for human experts to analyze digital pathology images. As mentioned above, the analysis of WSIs is a time-consuming procedure, including identifying and evaluating specific regions of interest within the tissue. The adoption of computer-assisted diagnosis models trained on clinical data in clinical workflows may help the pathologists in both tasks. The models can help identify possible regions of interest, thanks to the weights generated by the attention model, reducing the workload of pathologists and allowing them to focus on specific regions. The models can also evaluate the tissue within the identified regions of interest independently on their size (since they can classify single patches extracted from the images, cropped portions of WSIs or the entire WSI). Soon, computer-aided diagnosis systems trained on clinical data might thus help to reduce the workload of medical experts, allowing them to focus on the most crucial or uncertain findings and helping healthcare systems to increase the quantity and the quality of the diagnosis.

The third consequence of this work is that it paves the way to develop models that can more easily generalize to other clinical settings in the future, thanks to the application of the framework to larger cohorts of hospitals for training, considering the slightly lower, but still competitive, performance obtained in the publicly available datasets. Domain generalization (i.e. developing models that can perform good predictions on datasets different from those used for training the models) is one of the main factors preventing the translation of computational pathology algorithms to clinical settings. Increasing the size and the heterogeneity of training data can improve the performance and the generalization of the models, as it increases the variability in terms of tissue morphologies.

The models presented in this article show the capability to generalize, as demonstrated by the results obtained by testing them on 11,852 images from highly heterogeneous publicly available datasets, even though the overall performance is lower than the one obtained on private data (as shown in Table 2). Despite the fact that the performance on a few datasets can be considered good (such as cancer classification GlaS, CRC, TCGA-COAD, AIDA or low-grade dysplasia in UNITO), the overall slightly lower performance on publicly available datasets can be explained considering the variability of the acquisition procedure across centers (e.g. the staining variability, the whole slide scanners), the different meaning given to the classes (highlighted in the “CNN limitations” paragraph, method), such as normal, and the noise in weak labels that can be introduced by SKET (highlighted in the “SKET limitations” paragraph, Method).

The limitations that can reduce the capability to generalize on heterogeneous datasets can be solved in the future by considering the possibility to apply the approach using data from additional sources for training, including as well different algorithms to analyze the reports and the images, with several tissues and classes. The minimal efforts required to train the CNN and the possibility to endlessly increase the number of heterogeneous training data suggest that the proposed framework can be up-and-coming for digital pathology, as increasing training data is expected to improve performance and the capability to generalize.

This work sets a solid performance baseline for a methodology that can be translated to most diagnoses in future digital pathology. However, the components (such as the pre-processing algorithms and the data augmentation procedure) implemented in this paper can be changed and modified, according to the problem’s characteristics to solve and scientific advancements. Even though the paper focuses on the classification of five types of colon findings, the presented framework is not linked to a specific tissue or set of classes, and the authors are currently working on replicating the experiments on other tissue types and classes, where the framework is expected to work similarly (e.g. prostate, uterine cervix, etc.). Moreover, the proposed approach can be applied to other medical domains, such as Magnetic Resonance Imaging or Computed Tomography, and it can adopt different algorithms to extract the labels and to classify the images.

The overall aim of this work is to present the approach and the analysis methodology; therefore achieving high classification performance through finetuning was not among the objectives of the study. Performance can be improved by increasing the number of images/reports or exploiting more complex architectures, approaches and methods to handle the stain-variability of the images, as planned for future work.

In conclusion, the presented framework represents a breakthrough in the digital pathology domain. The framework paves the way for increasingly reliable computational pathology tools, with the critical advantages of being effective, capable of generalizing and capable of reducing to zero the human efforts to annotate extreme-scale data acquired in clinical routines. The code of SKET (https://github.com/ExaNLP/sket/) and MIL (https://github.com/ilmaro8/Multiple_Instance_Learning_instance_based) are publicly available.

## Methods

### Data from clinical pathology workflows

Data from Catania and Radboudumc hospitals are collected to assess the possibility to use data from clinical pathology workflow context, where data are heterogeneous, to train deep neural networks for computer-assisted diagnostics.

Data from clinical pathology workflows are not curated, allowing the simulation of the typical digital pathology workflow scenario, where it is often not possible to query the LIS for specific information about the WSIs. Therefore, as shown in Table 1, the classes are unbalanced reflecting another typical condition of LISs. In this case, data mainly include normal images. Data collected from clinical pathology workflows (Catania and Radboudumc) include 4419 colon WSIs and the associated diagnostic reports. The images are scanned with several scanners, leading to heterogeneous images. Images from Catania cohort with two Aperio scanners and two 3DHistech ones (at 20/40x), while images from Radboudumc hospital with a 3DHistech (at 40x). Furthermore, the images include different types of tissue samples: from Catania mainly colorectal polypectomies, biopsies, tissue resections and margin resections; while from Radboudumc mainly biopsies and few polypectomies.

### Pathology workflows data annotations

While the images used to train and validate the model are labeled with global labels (image-level annotations) a small subset of data is labeled with pixel-wise annotations, solely for evaluation purposes.

The pixel-wise annotations are a small percentage of tissue, including regions with tissue morphologies linked to the classes used as global labels. In the annotated images from Catania test partition (148/227), 52.73% of the tissue is annotated with one of the five classes presented in the paper, meaning that the rest of the tissue includes non-informative tissue or stroma (background is not included in the percentage). Considering each class, 4.62% (52/148 WSIs with local annotations) of tissue is annotated as cancer, 23.06% (44/148) with high-grade dysplasia, 10.57% (54/148) with low-grade dysplasia, 3.01% (23/148) with hyperplastic polyp and 11.28% with normal.

### Data from publicly available datasets

Data from publicly available repositories are collected to evaluate the CAD algorithms on highly heterogeneous images, to investigate how well the algorithm generalizes to heterogeneous medical centers.

This part of the data includes 11,888 images (WSIs and cropped sections of WSIs), collected from seven publicly available datasets: GlaS^36^, CRC^37^, UNITOPATHO^31,32^, TCGA-COAD^33^, Xu et al. colon dataset^38^, AIDA^34^ and IMP-CRC^35^. This partition is used to test the computer-assisted diagnosis algorithms in conditions of very high data heterogeneity. The images are scanned with several scanners and at several magnification levels, such as Zeiss MIRAX Midi (GlaS, 20x, 0.465 µm/pixel), Omnyz VL120 (CRC Dataset, 20x, 0.465 µm/pixel), Hamamatsu Nanozoomer S210 (UNITOPATHO, 20x, 0.46 µm/pixel), Hamamatsu Nanozoomer (Xu et al. colon dataset, 40x, 0.22 µm/pixel), Scanscope AT APERIO, Hamamatsu NanoZoomer XR, NanoZoomer XRL (AIDA, 20–40x, 0.50–0.25 µm/pixel), Leica GT450 (IMP-CRC, 40x, 0.25 µm/pixel). Furthermore, the subset of TCGA-COAD data is collected from nine medical centers.

### Pathology reports heterogeneity

Reports are highly-heterogeneous due to language, the report structure, the text input techniques used and the fact that different pathologists write the reports in separate timeframes. Language heterogeneity is related to the fact that reports from Catania are in Italian and the ones from Radboudumc are in Dutch. Report structure heterogeneity is related to the fact that reports have different fields. For instance, in Catania reports the field including the diagnosis refers to the entire WSI, while in Radboudumc reports the field including the diagnosis refers to the entire block of images from which the WSI originates. Finally, a further source of heterogeneity for reports is related to input methods. While pathologists manually type Catania reports, Radboudumc ones have been obtained using “speech to text” tools, thus introducing additional noise in the extraction process. Data are collected from pathology workflows without a preliminary visual inspection of the images.

### Images heterogeneity

**Fig. 5:**
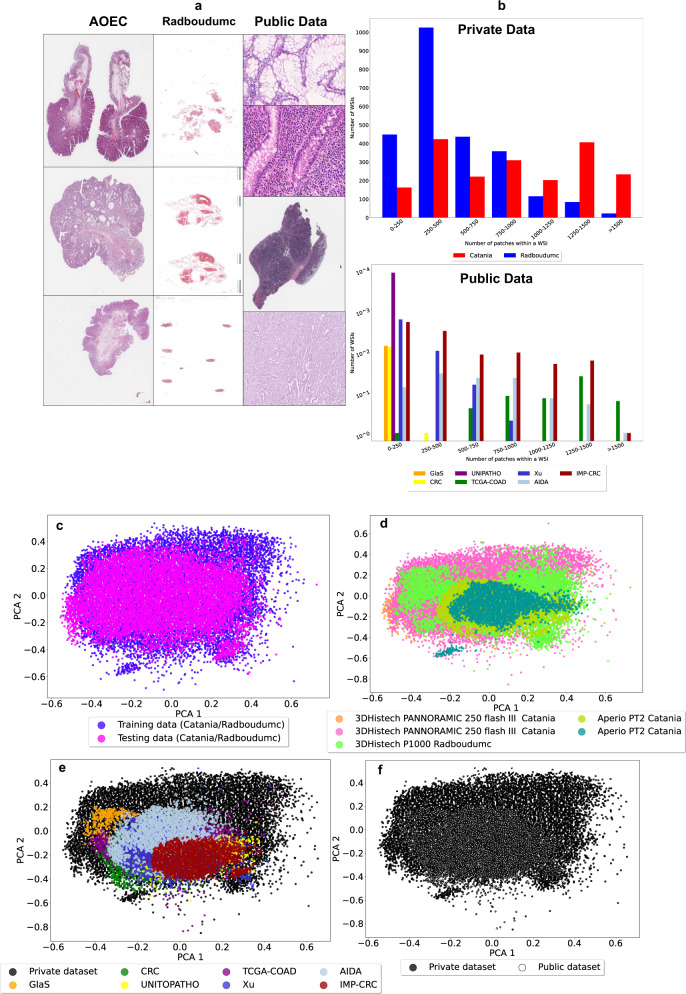
Overview of data heterogeneity. **a** Dataset includes different types of images: Catania partition includes biopsies, colorectal polypectomies and a few tissue resections (larger tissue samples), while Radboudumc includes biopsies and few colorectal polypectomies. **b** The different kind of image leads to a different number of patches per WSI. The upper histogram includes the number of WSIs as a function of the patches that they include, for Catania (red) and Radboudumc (blue). The fact that tissue resections and colorectal polypectomies are larger tissue samples than biopsies lead Catania to have larger images than Radboudumc. The lower histogram includes the number of images/WSIs as a function of the patches that they include, for GlaS (orange), CRC Dataset (yellow), UNITOPATHO (purple), TCGA-COAD (green), Xu dataset (light blue) and AIDA (celestial). **c** WSIs are scanned with several scanners, leading to heterogeneity in terms of colour. The heterogeneity is evaluated by analyzing the H&E matrices distributions, projected in two dimensions with Principal Component Analysis (PCA). The H&E matrix distributions for patches from training (purple) and testing (magenta) partitions in Catania and Radboudumc data. **d** The H&E matrix distributions for patches from pathology workflow, from APERIO PT2 (Catania, lime), APERIO PT2 (Catania, sky blue), 3DHistech PANNORAMIC 250 Flash III (Catania, pink), 3DHistech P1000 (Radboudumc, green). **e** The H&E matrix distributions for patches from private pathology workflow (black) and publicly available datasets (white). **f** The H&E matrix distributions for patches from GlaS (orange), CRC Dataset (yellow), UNITOPATHO (purple), TCGA-COAD (green), Xu dataset (light blue), AIDA (celestial) and pathology workflow (black).

Images considered in this work are heterogeneous in terms of sample type, size and colour. The images from pathology workflows include different types of samples: colorectal polypectomies, needle biopsies, tissue resections and cropped portions of WSIs (the latter one only on publicly available datasets). Tissue resections and colorectal polypectomies are usually more extensive than biopsies, leading to a highly variable number of patches, which are more numerous for Catania than for Radboudumc. The heterogeneity related to different tissue types is highlighted in Fig. 5a, b. Figure 5a shows a few examples of images coming from the datasets: the left column includes WSIs from Catania (three polypectomies), the central one from Radboudumc (three biopsies), and the right one from public datasets (tissue resection WSIs from TCGA-COAD and AIDA; cropped tissue sections from GlaS, CRC Dataset, UNITOPATHO colon dataset and Xu colon dataset). Figure 5b shows how different types of images lead to a different number of patches per image, considering data from pathology workflow (upper plot) and from publicly available datasets (lower plot). The histograms report the number of WSIs, including a corresponding range of patches.

Data from pathology workflow include an extensive range of patches mostly come from Catania (red), while data from publicly available datasets include cropped sections of WSIs (except for TCGA-COAD, AIDA and IMP-CRC), usually leading to images including a smaller number of patches (less than 500).

Images are highly heterogeneous in terms of stain variability because they originated from over ten centers and are acquired with over ten scanners (four scanners for the private data, seven for the public one) at magnification 20x or 40x. Stain variability is highlighted in Fig. 5a and Fig. 5c. Figure 5a includes images from the whole dataset. The left and the central columns include WSIs from digital pathology workflows, showing different shapes for different types of images. Stain variability related to different acquisition procedures (particularly scanners and chemical reagents) through medical centers is highlighted in Fig. 5–f. The Figures present the Hematoxylin & Eosin (H&E) colour distribution of the images, obtained projecting in two dimensions the H&E matrix (2 × 3), comparing the distribution of the stain distributions of both private and publicly available data. Figure 5c shows the data distribution from the private data, split in training and test partition. The lack of images selection during the data retrieval leads to a heterogeneous training partition. Figure 5d shows the distribution of data from the clinical workflows, split according to the scanner used to acquire the images. The subfigure shows how different scanners lead to different stain colours, even though the images come from the same medical center, as for Catania. Figure 5e compares data distribution from private digital pathology workflows and publicly available datasets. The choice to have private data acquired with several scanners allows having overlapping distributions with the publicly available datasets. Figure 5f shows the distribution of data from publicly available datasets. Most of the distributions do not overlap, highlighting the high stain variability within the test partition.

### Publicly available datasets class matching

Publicly available datasets are labeled with different classes than those used to train the model. To have a fair evaluation of the proposed CNN on public data, the predictions made by the presented CNN are mapped to the publicly available classes, as shown in Table 5.

**Table 5:** The mapping adopted on the publicly available datasets.

Dataset	Our classes	Original classes
GlaS^36^	Cancer, Hyperplastic polyp, Normal	Cancer, Benign (Hyperplastic polyp, Normal)
CRC^37^	Cancer, Hyperplastic polyp, Normal	Cancer, Benign (Hyperplastic polyp, Normal)
UNITOPATHO^31,32^	High-Grade Dysplasia, Low-Grade Dysplasia, Hyperplastic polyp, Normal	High-Grade Dysplasia, Low-Grade Dysplasia, Hyperplastic polyp, Normal
TCGA-COAD^33^	Cancer	Cancer
Xu^38^	Cancer, Normal	Cancer, Normal
AIDA^34^	Cancer, High-grade Dysplasia, Low-Grade Dysplasia, Hyperplastic polyp, Normal	Cancer, Dysplasia (High-grade Dysplasia, Low-Grade Dysplasia), Hyperplastic polyp, Normal
IMP-CRC^35^	Cancer, High-grade Dysplasia, Low-Grade Dysplasia, Hyperplastic polyp, Normal	High-risk (Cancer & High-grade Dysplasia), Low-grade dysplasia, Non-neoplastic (Hyperplastic polyp & Normal)

### Automated extraction of image labels from free-text diagnostic reports

SKET adopts a combination of pre-trained Named Entity Recognition (NER) models and unsupervised Entity Linking (EL) methods to extract key concepts (entities) from the diagnostic reports and to link them to the reference ontology (https://w3id.org/examode/ontology/). The use of pre-trained NER models and unsupervised EL methods makes SKET suitable for weak supervision tasks. SKET consists of three components: (1) Named Entity Recognition, (2) Entity Linking, and (3) Data Labeling.

To perform Named Entity Recognition, SKET relies on a combination of pre-trained neural models and rule-based techniques. Specifically, it adopts and extends ScispaCy models^44^ (e.g. the “en_core_sci_lg” model), which provide full NER pipeline for biomedical data, with a large medical vocabulary, and 600,000 Word2Vec^45^ word vectors trained on the PubMed Central Open Access Subset^46^. In this regard, SKET can be deployed with any of the *core* models available at: https://allenai.github.io/scispacy/.

Regarding Entity Linking, SKET employs a combination of ad-hoc and similarity matching techniques to link the extracted entities to unique concepts within the reference ontology. Given an extracted entity, SKET first tries to match it using ad-hoc matching and when it fails SKET employs similarity matching.

As for Data Labeling, SKET performs a mapping from the linked concepts to a set of annotation classes. The annotation classes are (i) Cancer; (ii) High-grade dysplasia; (iii) Low-grade dysplasia; (iv) Hyperplastic polyp; and, (v) Normal.

Although SKET does not include trainable parameters, the ad-hoc matching techniques have been tuned using data coming from the digital pathology workflow of the two hospitals. These data include independent samples (around 200 from Catania and around 200 from Radboudumc), not used for training, validating or testing the CNN, nor for testing SKET. The ad-hoc rules developed to match concepts using these independent samples have also been verified by pathologists. Furthermore, such data has been used to check the viability of using translation models without injecting noise into SKET components.

### Image pre-processing

The images are pre-processed with the same approach, regardless of the source. The approach involves splitting the images in patches and selecting the ones coming from tissue regions, discarding regions from the background.

WSIs splitting is necessary to the gigapixel nature of WSIs, since modern GPUs hardware has limited memory and cannot handle large images. Images are split into patches of 224 × 224 pixels, extracted from magnification 10x, using Multi_Scale_Tools library^47^. The patch size is chosen considering that the ResNet34 backbone used as CNN requires this input data size. The magnification level is chosen considering that the WSIs at 10x allow visualizing the components that correctly identify the considered classes. Patches coming from background regions are not considered, since they are not informative for the tissue analysis. The identification of tissue regions and background regions is performed by generating tissue masks with HistoQC tool^48^.

### Data augmentation

During the training, data augmentation is applied to the input data, at WSI-level. The data augmentation pipeline is implemented with albumentations library^49^ and it includes three operations: rotation, horizontal and vertical flipping and colour augmentation. For each WSI, a transformation pipeline is generated, using the operations with a probability of 0.5. The pipeline is applied at WSI-level, so that the same combination of transformations is applied to each patch within a WSI.

### MIL algorithm

The CNN is trained using a Multiple Instance Learning (MIL) framework, trained at instance-level.

MIL^16,22,23,50,51^ is a weakly-supervised framework that allows facing problems where data are organized as a bag of instances^51^^,^^52^ and the information available on the data regards the entire bag, without any local information about the instances. The framework is based on the MIL assumption^52^, which coordinates the relationship between the bag and the instances. The original MIL assumption asserts that a bag is positive if it includes at least one positive instance, while it is negative if it does not include any positive instance. The assumption can be relaxed to be adopted in problems where the bag is identified by the distribution of its patches, such as multiclass problems. A MIL problem can be formulated at two levels^51^: the bag-level and the instance-level. In both levels, the component that aggregates the instance features or the instances predictions is called pooling algorithm. Multiple Instance Learning fits with the requirements of weakly-annotated WSIs analysis, due to the characteristics of the images, showing an increasing number of applications yearly developed and published^16^. Histopathology image classification can be formulated as a MIL problem, where a WSI represents a bag *X*_*n*_ that includes *p* patches and the information available on the data regards the entire WSI, as in the MIL CNN presented in this paper.

The MIL CNN presented in this paper produces predictions for the single instances. The model includes several components, as shown in Fig. 1: a pre-trained convolutional backbone (ResNet34), an intermediate fully-connected layer, a classifier and an attention network. The activation function between the intermediate layer and the classifier is a ReLU. The convolutional backbone is pre-trained with MoCo v2 algorithm^53^ (presented in CNN pre-training section) and it is frozen during the training. The intermediate fully-connected layer produces smaller feature vectors, called embeddings from the ResNet features, for each patch within a WSI. The classifier produces predictions from the patch embeddings. The attention network is a pooling layer (state-of-the-art algorithm), that aggregates the predictions for the single patches to have a global prediction for the WSI^22,54^. The attention network gives a weight to each patch, depending on their importance in the global predictions. The sum of the weight is 1. Considering that the global WSI prediction is not the sum of the single predictions/embeddings, the attention pooling layers allow having a learnable function to aggregate the embeddings or the predictions.

The choice of training a MIL instance-based framework shows advantages and disadvantages. The most important advantage is that this kind of CNN can be used in clinical settings, showing to the pathologist the predictions of the model for each patch. Furthermore the attention pooling layer may underline some regions of interest for the pathologist. The most crucial disadvantage is that the model’s performance at WSI (bag) level is lower than MIL embedding-based frameworks^51^. The research articles involving MIL are constantly increasing, targeting several critical aspects, such as the pooling layer (Ilse et al.^22^) and strategies to identify the most relevant patches to use for classification (Lu et al.^23^). Therefore, in the long-term, it can easily allow solving the WSI classification problem with high performance. Anyway, in the current state-of-the-art, it is a reasonable, straightforward and promising algorithm to face the challenges described in the article.

### CNN pre-training

The CNN backbone (ResNet34) is pre-trained using MoCo v2^53^, a self-supervised algorithm, adopted to pre-train CNN to learn features related to input data.

Pre-training CNN backbones is a standard approach to overcome the current limitations of MIL algorithms, which require large amounts of memory, that may easily exceed modern hardware capabilities. Typically, MIL CNN backbones are pre-trained on ImageNet and then frozen, thus reducing training to fully-connected layers only. However, ImageNet dataset includes natural images, so the pre-trained weights are trained to learn feature that might not be suited to be used on solving computational pathology tasks. To avoid this drawback, we pre-trained our CNN backbone (ResNet34) using MoCo v2^53^, a self-supervised algorithm that allows to pre-train deep neural networks by learning features related to the input data. The application of MoCo to CNN showed higher performance in several tasks, compared with the same network using ImageNet weights^53^.

From a technical point of view, MoCo is a contrastive self-supervised algorithm, that trains the network to learn similarities and dissimilarities between input data. Given the unsupervised nature of the algorithm, the input data do not require to be labeled. The similarity/dissimilarity relationships between input data are obtained using data augmentation. Each sample (i.e. a patch) in a batch is augmented, under the hypothesis that augmented versions are similar to each other and dissimilar to the other inputs of the batch. Augmented versions of input samples are stored in a queue, that is used to retrieve dissimilar examples. The data augmentation pipeline includes several operations, reported in Table 6, and it is implemented using Albumentation library^49^. The operations are applied for each input samples, with a probability of 0.8.

**Table 6:** Operations involved in the MoCo pre-training.

Operation	Parameters
Horizontal and vertical flipping	–
Random rotations	90, 180, 270 degress
Hue saturation value	Hue_limit (−20, 20), Saturation_limit (−30, 20), Value limit (−20, 20)
RGBShift	R_shift (−20,10), G_shift (−20, 10), B_shift (−20, 10),
CLAHE	Clip_limit = 1.0, tile_grid_size = (4,4)
Random brightness	Limit = 0.2
Random contrast	Limit = 0.2
Gaussian noise	Limit (10, 50), mean = 0
Elastic transformation	Alpha = 1, sigma = 30, alpha_affine = 30
Grid distorsion	Num_steps = 3, distort limit = 0.3
GlassBlur	Sigma = 0.1, max_delta = 1, iterations = 1
Optical distorsion	distort limit = 0.3, shift_limit = 0.3

On top of this, during the training of MoCo, a H&E-invariant^43^ optimization is applied to the CNN, to learn features invariant to stain. Considering the stain variation across centers, the adoption of this approach may allow to increase the generalization of the CNN in data collected from different and heterogeneous centers. The network is trained with a batch size of 256 and queue including 16,384 samples.

### K-fold cross-validation

The CNN is trained and validated using a k-fold cross-validation approach to demonstrate that the model is robust to the selected training data.

Training data are split into k (in this case *k* = 10) groups, so that in each training the data from k-1 groups are used to train the CNN and data from the other group are used to validate it. The split is made at the patient level to avoid shared images between training and validation partitions. Finally, the CNN is evaluated on the test partition, reporting the average and the standard deviation of the k models.

### Hyperparameters

The hyperparameters used to train the model are optimized using a grid search algorithm^55^. The grid search algorithm aims to identify the optimal configuration of CNN hyperparameters. In this case, the optimal configuration allows the CNN to reach the lowest loss function on WSI classification, on the validation partition. The grid search involves several hyperparameters: the number of epochs for training the model (15, after this number of epochs the model does not reach a lower loss function), the optimized (Adam; SGD and Adam were tested), the learning rate (10^−3; 10^−2, 10^−3,10^−4, 10^−5 were tested), the decay rate (10^−3; 10^−2, 10^−3,10^−4, 10^−5 were tested) and the number of nodes within the embedding layer before the classifier (128; 32,64,128,256 were tested).

### Metrics used to evaluate the model

The performance of the model is evaluated at patch-level and WSI-level.

At patch-level, the classification is a multiclass problem and the model is evaluated using Cohen’s κ-score. Cohen’s κ-score measures the agreement between raters. It is usually adopted in scientific literature to evaluate the agreement between pathologists. In this case, it measures the agreement between the model predictions and the ground truth. The metric varies between −1 (complete disagreement) and 1 (complete agreement). Cohen’s κ-score = 0 means agreement by chance.

At WSI-level and image-level, the classification is a multilabel problem and the model is evaluated using the micro-average of accuracy and the weighted macro F1-score to tackle the class unbalance. Accuracy is the fraction of correct predictions (true positives + true negatives) on the total number of the predictions and varies between 0 (total wrong predictions) and 1 (perfect predictions). Being the task proposed a multilabel classification, the accuracy metric is averaged using micro-accuracy, working with the single true positives, etc. F1-score is the average between the precision and the recall. The metric is averaged using the macro-weighted average, to tackle the class unbalance of the dataset. The macro-weighted average evaluates the F1-score separately for each class and uses a weight related to the number of true labels of each class (support). Furthermore, the single class performance is evaluated using recall and precision, where the precision measures the ability of the classifier not to label negative samples as positive ones and the recall is the ability of the model to classify all the positive samples correctly.

### Feature space

The feature space is visualized, reducing the embedding layer’s output (128 elements) with the t-distributed stochastic embedding (t-SNE) in two dimensions. The reduction is applied to the patches of the test partition, where the predictions of the CNN are greater than 0.5.

### Software & hardware

The whole pipeline is implemented with several Python libraries.

Pytorch 1.1.0 is used to model, train and test the CNNs. Openslide 3.4.1 and ASAP 1.9 are used to access the WSIs. Scikit-learn 0.23.1 is used to evaluate the performance metrics of the models. Albumentations 1.8 is used for implementing the data augmentation pipeline.

All the experiments are executed on a Tesla V100 GPU.

### SKET performance and limitations

The SKET pipeline shows high-accurate performance on both Catania and Radboudumc data, even though the pipeline can still be improved in terms of report annotations.

Table 7 reports the single-class performances in both Catania and Radboudumc data.

**Table 7:** Overview of SKET performance, reporting the precision, the recall and the F1-score of the single classes, in both Catania and Radboudumc reports.

Class	Precision	Recall	F1-score	Support
SKET performance per class (Catania)				
Cancer	0.84	0.94	0.89	379
High-Grade Dysplasia	0.90	0.92	0.91	454
Low-Grade Dysplasia	0.75	0.90	0.82	529
Hyperplastic polyp	0.68	0.94	0.79	181
Normal glands	0.87	0.92	0.89	438
SKET performance per class (Radboudumc)				
Cancer	0.94	0.95	0.94	188
High-Grade Dysplasia	0.66	0.83	0.73	94
Low-Grade Dysplasia	0.85	0.81	0.83	453
Hyperplastic polyp	0.84	0.97	0.90	428
Normal glands	0.92	0.88	0.90	1048

The support of the class represents the number of true positive cases for a particular class.

On Catania data, SKET achieves higher recall values (all the classes over 0.9) than precision ones (all classes under 0.90). In particular, low-grade dysplasia and hyperplastic polyp show low precision scores with values equal to 0.75 and 0.68, respectively. We performed a failure analysis to investigate the situation by checking the samples where SKET labels are wrong. For what concerns the low-grade dysplasia class, the false positives predicted by SKET may be linked to the keyword ‘dysplasia’, which is also used for the class ‘high-grade dysplasia’ and to the adjectives used to describe it (e.g. ‘severe’). For what concerns hyperplastic polyp, the annotations include several false positives due to the presence of the following sentence: “margin of resection on hyperplasia-adenomatous mucosa”. In this case, the concept of hyperplasia describing the resection margin leads SKET to annotate the sample as hyperplastic polyp. However, after a revision of the reports by pathologists, this concept must be interpreted as the absence of hyperplastic polyps.

On Radoudumc data, SKET achieves high precision and recall scores for all the classes, leading to a F1-score that is over 0.83 for all classes, except for high-grade dysplasia. The high precision score highlights the ability of SKET to avoid false positives. The only class that shows low performance in precision is high-grade dysplasia. The large number of high-grade dysplasia false positives can be explained by considering the reports mislabeled as high-grade dysplasia. In most of these reports, the absence of the concept (e.g., ‘NO high-grade dysplasia’) is explicitly written, but SKET erroneously identifies the ‘high-grade dysplasia’ keyword as a class and thus mislabels the report. Another problem is related to the keyword ‘severe’, that can be used to describe the dysplasia condition (i.e. high-grade dysplasia). However, the adjective ‘severe’ may be used to describe other conditions as well, such as cancer. On the other hand, the recall scores—although over 0.81 for all the classes—suggest that SKET misses few relevant concepts.

The different behavior of SKET in the two datasets can be attributed to the different medical language and style of the Catania and Radboudumc reports. In this regard, Catania reports include several words and details that can be misinterpreted by SKET. On the other hand, Radboudumc reports are more concise and precise. Another important outcome to stress is the high performance obtained by SKET on normal samples. In addition to those reports where normal glands are mentioned, this class is also adopted when none of the entities identified in the reports matches one of the considered classes. Therefore, SKET annotates reports with ‘normal’ class when it does not identify any of the other classes.

Aside from class-specific problems, SKET can be further improved by working on a few general-level issues. For instance, SKET can fail when reports specify the absence of a given concept—suggesting that we need to improve its ability to detect negations within text. Furthermore, SKET might fail to split blocks-level reports appropriately, ending up considering concepts related to different sets of images. Nevertheless, the high recall achieved for each class in both datasets suggests that SKET identifies positive examples with high confidence.

### CNN performance and limitations

The results obtained, contextualized in the field of colon histopathological images diagnosis, show: (i) high performance in WSI classification on Catania and Radboudumc datasets; (ii) the capability to generalize to unseen data from publicly available datasets; (iii) moderate performance at patch-level. Such performance can be further improved by increasing the number of images/reports (for instance by relying on several medical sources) or through the exploitation of more complex architectures and approaches, as planned for future work.

As described in the main part of the text, the overall performance on the Catania test partition is high (micro-accuracy = 0.91) but the situation gets more complex when looking at single classes. Table 8 reports the single-class performance in both Catania and Radboudumc data.

**Table 8:** Overview of CNN performance, reporting the precision, the recall and the F1-score of the single classes for image-level classification, in both Catania and the publicly available datasets.

Class	Precision	Recall	F1-score	Support
CNN performance per class (Catania)				
Cancer	0.889 ± 0.042	0.749 ± 0.065	0.809 ± 0.029	52
High-grade dysplasia	0.712 ± 0.081	0.648 ± 0.116	0.681 ± 0.047	44
Low-grade dysplasia	0.595 ± 0.023	0.850 ± 0.066	0.700 ± 0.012	54
Hyperplastic polyp	0.854 ± 0.140	0.513 ± 0.146	0.612 ± 0.102	23
Normal	0.928 ± 0.064	0.982 ± 0.016	0.954 ± 0.034	79
CNN performance per class (Radboudumc)				
Cancer	0.826 ± 0.069	0.540 ± 0.105	0.642 ± 0.067	50
High-grade dysplasia	0.896 ± 0.106	0.145 ± 0.053	0.245 ± 0.077	22
Low-grade dysplasia	0.838 ± 0.032	0.613 ± 0.075	0.704 ± 0.043	92
Hyperplastic polyp	0.717 ± 0.085	0.726 ± 0.083	0.713 ± 0.033	62
Normal	0.870 ± 0.012	0.821 ± 0.033	0.844 ± 0.018	219
CNN performance per class (GlaS)				
Cancer	1.0 ± 0.0	0.625 ± 0.087	0.766 ± 0.065	91
Normal	0.561 ± 0.068	1.0 ± 0.0	0.717 ± 0.049	42
CNN performance per class (CRC)				
Cancer	0.857 ± 0.044	0.896 ± 0.041	0.874 ± 0.011	69
Normal	0.904 ± 0.030	0.859 ± 0.057	0.879 ± 0.019	71
CNN performance per class (UNITO sections)				
High-grade dysplasia	0.504 ± 0.050	0.210 ± 0.051	0.293 ± 0.052	1370
Low-grade dysplasia	0.820 ± 0.015	0.641 ± 0.042	0.719 ± 0.023	5804
Hyperplastic polyp	0.279 ± 0.041	0.435 ± 0.077	0.332 ± 0.022	544
Normal glands	0.290 ± 0.026	0.533 ± 0.056	0.375 ± 0.030	950
CNN performance per class (UNITO WSIs)				
High-grade dysplasia	0.722 ± 0.173	0.182 ± 0.085	0.279 ± 0.105	46
Low-grade dysplasia	0.789 ± 0.021	0.923 ± 0.027	0.850 ± 0.009	184
Hyperplastic polyp	0.871 ± 0.048	0.590 ± 0.152	0.688 ± 0.107	41
Normal glands	0.560 ± 0.079	0.776 ± 0.064	0.646 ± 0.052	21
CNN performance per class (TCGA-COAD)				
Cancer	1.0 ± 0.0	0.862 ± 0.051	0.926 ± 0.029	50
CNN performance per class (Xu)				
Cancer	0.685 ± 0.074	0.828 ± 0.058	0.746 ± 0.035	355
Normal	0.785 ± 0.051	0.609 ± 0.132	0.677 ± 0.084	362
CNN performance per class (AIDA)				
Cancer	0.682 ± 0.106	0.835 ± 0.046	0.744 ± 0.056	31
Low-grade dysplasia	0.590 ± 0.097	0.50 ± 0.000	0.537 ± 0.044	4
Hyperplastic polyp	0.0 ± 0.0	0.0 ± 0.0	0.0 ± 0.0	1
Normal	0.858 ± 0.013	0.748 ± 0.093	0.796 ± 0.052	65
CNN performance per class (IMP-CRC)				
Cancer & HGD	0.570 ± 0.049	0.856 ± 0.044	0.681 ± 0.027	268
Low-grade dysplasia	0.851 ± 0.038	0.713 ± 0.094	0.770 ± 0.048	547
Hyperplastic & normal	0.695 ± 0.072	0.546 ± 0.069	0.605 ± 0.037	271

The support of the class represents the number of true positive cases for a particular class.

The CNN shows high performance on cancer and normal images from Catania test partition, paving the way to build effective tools for screening settings in hospitals. However, on the other three classes, the model has some limitations. In the Catania dataset, the method shows a precision over 0.70 for each class, except for low-grade dysplasia (0.61). This problem derives from the fact that the low-grade dysplasia class is linked to gland morphology, meaning that it can be easily mistaken for a normal tissue patch. Similarly, both high-grade dysplasia and hyperplastic polyps exhibit similar morphologies to low-grade dysplasia, which makes the classification problem particularly hard. On top of this, the training dataset includes 249 samples (from Catania cohort) annotated with both high-grade and low-grade dysplasia. Another motivation for the low precision of low-grade dysplasia in Catania data relates to the unbalance of the dataset, where low-grade dysplasia is the most occurring class. Because of this, the CNN may be prone to overfit on this class, predicting low-grade dysplasia more often than required. Low-grade dysplasia also shows low recall (0.51), confirming the prediction. As described in the main part of the text, the overall performance on the Radboudumc test partition is high (micro-accuracy = 0.90) but the situation gets more complex when looking at single classes. The performance on Radboudumc test partition shows a similar situation to what shown for Catania: the model achieves high performance in normal cases prediction, but lower performance in the other classes (F1-score around 0.60 for cancer, low-grade and hyperplastic polyp). As mentioned above, this fact is a promising outcome, since it makes the model suitable for screening purposes. However, the performance on high-grade dysplasia for Radboudumc data needs to be improved (F1-score = 0.24). While the precision for the class is high (0.89), the recall is very low (0.14). Therefore, the model does not identify the presence of high-grade dysplasia in most of Radboudumc slides. This limitation can be explained by considering several aspects. The first aspect involves the low number of high-grade dysplasia cases included in the training dataset, which makes it the least represented class. The second aspect involves the large number of cases including cancer and dysplasia tissues in the test partition. Cancer and low-grade dysplasia show similar tissue morphologies to high-grade dysplasia. Therefore, the CNN might be prone to predict cancer and low-grade dysplasia instead of high-grade dysplasia in Radboudumc partition samples. The third aspect that explains the limited performance on high-grade dysplasia involves the small regions including high-grade dysplasia tissue within the WSIs. Most of the reports manually annotated with high-grade dysplasia include the keyword ‘focal’—which suggests that the portion of high-grade dysplasia is limited or describe unclear findings—, or the phrases ‘from moderate to severe dysplasia’ and ‘from severe dysplasia to carcinoma’—which suggest that the morphology is not well defined even for pathologists.

In the publicly available datasets, the overall performance shows good results, but still slightly lower in general, comparing it with the Catania and Radboudumc test partitions. This problem might be linked to several factors: the different meaning given to the classes (such as normal or high-grade adenoma), the low inter-pathologist agreement (even among humans) and the different acquisition procedures used to digitize the samples.

The class mapping proposed aims to partially alleviate the different meanings of the classes (e.g. normal and benign) during the evaluation of the models, allowing to have a fair evaluation of the model on external datasets. However, even though the mapping allows the evaluation of the model on external datasets, the features learned by the CNN are not directly optimized on those classes and concepts (e.g. benign class).

The low inter-pathologist agreement evaluation is a well-known problem in digital pathology (Cohen’s κ-score = 0.54-67), leading to a highly variable ground truth. An example of the different meaning given to the classes may identified in the CNN performance on normal samples.

The heterogenous acquisition procedures across medical centers may contribute to the slighlty lower performance reached by the CNN on publicly available datasets, compared with the performance on internal test partition. In particular, the performance on publicly available datasets shows lower recall and precision (than the scores achieved for Catania and Radboudumc), meaning that the model predicts more false positives and false negatives. The UNITOPatho dataset performance shows both the problems related to false positives and false negatives. This dataset is evaluated twice: at regions of interest level and at WSI-level. At regions of interest level, the precision of hyperplastic polyp and normal tissue is under 0.3, but at WSI-level the same classes show a precision of 0.87 and 0.56 respectively. Furthermore, the recall reached in the dataset (both the sections and the WSIs evaluation) is below 0.21, while in the other datasets the recall is over 0.5. One hypothesis to explain the difference in performance is that the model is trained and optimized on WSIs and not on small regions of interest. However, since on GlaS and CRC, the CNN shows high level of precision, the problem might be linked to other reasons too, such as fuzzy annotation on the single regions of interest.

The mentioned problems (the different meaning given to the classes, the high-variable image ground truth and the data heterogeneity) may be alleviated considering several options. One option may be to adopt a different CNN architecture, to better distinguish between the tissue morphologies. The approach presented in this article does not require a particular computer vision algorithm, allowing using other methods. Another option may be to include additional images, in particular for the less represented classes, collecting data including rare conditions, even if, due to their limited number may include a low tissue variability, reducing the model capability to generalize on heterogeneous data.

### Reporting summary

Further information on research design is available in the Nature Research Reporting Summary linked to this article.

## Supplementary information

Reporting Summary

## Data Availability

The dataset includes data from private and publicly available datasets. Private data are collected from Catania cohort (Azienda Ospedaliera Cannizzaro and Gravina Hospital Caltagirone ASP, Catania, Italy) and the Radboud Medical University Center (Radboudumc, Nijmegen, The Netherlands). The WSIs are scanned with several scanners: images from Catania hospital with two Aperio scanners and two 3DHistech ones (at 20/40x), while images from Radboudumc hospital with a 3DHistech (at 40x). Data from Catania mainly include colorectal polypectomies, biopsies and few tissue resections, while data from Radboudumc mainly include biopsies and few polypectomies. We are currently evaluating together with the clinical partners if it is possible to release the clinical data from a private data as an open access dataset, according to ethics guidelines of the involve committees and European and national law. Publicly available data include images from six datasets: GlaS^36^, CRC^37^, UNITOPATHO^31^, TCGA-COAD^33^, Xu et al. colon dataset^38^ and AIDA^34^, IMP-CRC^29^. The images are scanned with several scanners and at several magnification levels, such as Zeiss MIRAX Midi (GlaS, 20x), Omnyz VL120 (CRC Dataset, 40x), Hamamatsu Nanozoomer S210 (UNITOPATHO, 20x), Hamamatsu Nanozoomer (Xu et al. colon dataset, 40x), Scanscope AT APERIO, Hamamatsu NanoZoomer XR and NanoZoomer XRL (AIDA, 20–40x). Data are publicly available on a webpage of the organization that collected them, except for AIDA, Xu dataset and IMP-CRC that are available upon request. GlaS (https://warwick.ac.uk/fac/cross_fac/tia/data/glascontest/). CRC (https://warwick.ac.uk/fac/cross_fac/tia/data/crc_grading/). UNITOPATHO (https://ieee-dataport.org/open-access/unitopatho). TCGA-COAD (https://portal.gdc.cancer.gov/projects/TCGA-COAD). Xu et al. (https://bmcbioinformatics.biomedcentral.com/articles/10.1186/s12859-017-1685-x). AIDA (https://datahub.aida.scilifelab.se/10.23698/aida/drco). IMP-CRC (https://www.nature.com/articles/s41598-021-93746-z#data-availability). The list of TCGA-COAD image ids is uploaded in the Github repository.

## Code availability

The code for SKET (https://github.com/ExaNLP/sket/) and MIL (https://github.com/ilmaro8/Multiple_Instance_Learning_instance_based) are publicly available. The model weights of the best MIL CNN are uploaded in the repository.
