# Supplementary material for: Unleashing the potential of digital pathology data by training computer-aided diagnosis models without human annotations
Source: NPJ Digit Med. 2022 Jul 22;5:102. doi: 10.1038/s41746-022-00635-4 (PMC9307641; doi:10.1038/s41746-022-00635-4)
Supplement: Supplementary file 1 — Reporting Summary [file 41746_2022_635_MOESM1_ESM.pdf]

nature portfolio

Corresponding author(s): Niccolò Marini

Last updated by author(s): Oct 20, 2021

Reporting Summary

Nature Portfolio wishes to improve the reproducibility of the work that we publish. This form provides structure for consistency and transparency in reporting. For further information on Nature Portfolio policies, see our Editorial Policies and the Editorial Policy Checklist.

Statistics

For all statistical analyses, confirm that the following items are present in the figure legend, table legend, main text, or Methods section.

n/a	Confirmed	
☐	☒	The exact sample size (n) for each experimental group/condition, given as a discrete number and unit of measurement
☒	☐	A statement on whether measurements were taken from distinct samples or whether the same sample was measured repeatedly
☒	☐	The statistical test(s) used AND whether they are one- or two-sided<br>*Only common tests should be described solely by name; describe more complex techniques in the Methods section.*
☒	☐	A description of all covariates tested
☒	☐	A description of any assumptions or corrections, such as tests of normality and adjustment for multiple comparisons
☒	☐	A full description of the statistical parameters including central tendency (e.g. means) or other basic estimates (e.g. regression coefficient) AND variation (e.g. standard deviation) or associated estimates of uncertainty (e.g. confidence intervals)
☒	☐	For null hypothesis testing, the test statistic (e.g. F, t, r) with confidence intervals, effect sizes, degrees of freedom and P value noted<br>*Give P values as exact values whenever suitable.*
☒	☐	For Bayesian analysis, information on the choice of priors and Markov chain Monte Carlo settings
☒	☐	For hierarchical and complex designs, identification of the appropriate level for tests and full reporting of outcomes
☒	☐	Estimates of effect sizes (e.g. Cohen's d, Pearson's r), indicating how they were calculated

Our web collection on statistics for biologists contains articles on many of the points above.

Software and code

Policy information about availability of computer code

Data collection	No specific software were used to collect the data.
Data analysis	The code is written in python 3. Openslide 3.4.1 is used to access the whole slide images, pytorch 1.7.0 to train deep learning methods,

For manuscripts utilizing custom algorithms or software that are central to the research but not yet described in published literature, software must be made available to editors and reviewers. We strongly encourage code deposition in a community repository (e.g. GitHub). See the Nature Portfolio guidelines for submitting code & software for further information.

Data

Policy information about availability of data

All manuscripts must include a data availability statement. This statement should provide the following information, where applicable:
- Accession codes, unique identifiers, or web links for publicly available datasets
- A description of any restrictions on data availability
- For clinical datasets or third party data, please ensure that the statement adheres to our policy

Private data (including whole slide images and medical reports) are not publicly available. The authors are planning to release them in the future. The external test set is publicly available.

Field-specific reporting

Please select the one below that is the best fit for your research. If you are not sure, read the appropriate sections before making your selection.

☒ Life sciences ☐ Behavioural & social sciences ☐ Ecological, evolutionary & environmental sciences

For a reference copy of the document with all sections, see nature.com/documents/nr-reporting-summary-flat.pdf

Life sciences study design

All studies must disclose on these points even when the disclosure is negative.

Sample size	Data are collected from private databases of two hospitals (Cannizzaro Hospital, Catania, Italy; Radboud University Medical Center, Nijmegen, Netherlands). As usually performed in AI studies, the study was performed choosing the largest possible training set. The samples are selected to have as much as possible balanced disease classes. In this particular case, the size of the test set was carefully chosen too to included as many testing cases as possible.
Data exclusions	A part of the proprietary data was excluded to obtain balanced disease classes distributions. Within each disease class, the removed cases selection was randomized
Replication	The deep learning algorithms were trained ten times and the average/std deviation are reported, for each result.
Randomization	Random samples were chosen among the data available for each class. The allocation of samples in training, validation and testing partitions involved three rules: 1) testing partition includes data manually annotated by pathologist; 2) data coming from a specific patient cannot be in two different partitions; 3) partitions should be as much balanced as possible in terms of class distributions.
Blinding	The experiments are based on digitized pathology slides and reports. The training set the testing set are inherently blinded as they come from different datasets.

Reporting for specific materials, systems and methods

We require information from authors about some types of materials, experimental systems and methods used in many studies. Here, indicate whether each material, system or method listed is relevant to your study. If you are not sure if a list item applies to your research, read the appropriate section before selecting a response.

Materials & experimental systems

n/a	Involved in the study	
☒	☐	Antibodies
☒	☐	Eukaryotic cell lines
☒	☐	Palaeontology and archaeology
☒	☐	Animals and other organisms
☐	☒	Human research participants
☐	☒	Clinical data
☒	☐	Dual use research of concern

Methods

n/a	Involved in the study	
☒	☐	ChIP-seq
☒	☐	Flow cytometry
☒	☐	MRI-based neuroimaging

Human research participants

Policy information about studies involving human research participants

Population characteristics	Digitized images from (Cannizzaro Hospital, Catania, Italy; Radboud University Medical Center, Nijmegen, Netherlands) were digitized in the last 10 ten years.
Recruitment	No patient recruitment was needed. All clinical data were collected from the Laboratory Information Systems.
Ethics oversight	Ethics approval was obtained by the competent institutions at the Cannizzaro Hospital (Catania, Italy) and the Radboud University Medical Center (Nijmegen, Netherlands).

Note that full information on the approval of the study protocol must also be provided in the manuscript.

Clinical data

Policy information about clinical studies

All manuscripts should comply with the ICMJE guidelines for publication of clinical research and a completed CONSORT checklist must be included with all submissions.

Clinical trial registration	This is not a clinical trial, but a study based on clinical data. Ethics approval was obtained by the competent institutions at the

Clinical trial registration	Cannizzaro Hospital (Catania, Italy) (dossier number 4428 of 12/12/2018) and the Radboud University Medical Center (Nijmegen, Netherlands) (dossier number 2018-4764).
Study protocol	The study protocol is described in the article.
Data collection	WSIs from private dataset were digitized with two Leica Aperios AT2, 3DHistech PANNORAMIC 250 Flash III and 3DHistech P1000.
Outcomes	The outcomes are detailed in the article. In brief, AI based computer diagnostic tools are obtained with no human supervision from clinical data and evaluated on external test data.
